# Mitophagy in ophthalmic pathologies: Molecular mechanisms and therapeutic implications

**DOI:** 10.1097/MD.0000000000047600

**Published:** 2026-05-08

**Authors:** Yibo Han, Jiaojiao Feng, Xiaoqi Gong, Guodong Tang, Yixue Yin, Jing Li, Yuxi Liu, Jun Zhang, Jike Song, Hongsheng Bi

**Affiliations:** a School of Ophthalmology and Optometry, Shandong University of Traditional Chinese Medicine, Jinan, China; b Department of Scientific Research, Affiliated Eye Hospital of Shandong University of Traditional Chinese Medicine, Jinan, China; c Department of Scientific Research, Shandong Key Laboratory of Integrated Traditional Chinese and Western Medicine for Prevention and Therapy of Ocular Diseases, Jinan, China.

**Keywords:** age-related macular degeneration (AMD), diabetic retinopathy (DR), glaucoma, mitophagy, oxidative stress, regulation of autophagy

## Abstract

Mitophagy, a selective autophagic process responsible for the degradation of dysfunctional mitochondria, serves as a critical regulator of cellular homeostasis. Despite its emerging significance in ocular pathophysiology, comprehensive analyses bridging molecular mechanisms to clinical translation remain scarce. The retina, with its high metabolic demands and reliance on mitochondrial bioenergetics, is particularly vulnerable to mitophagic dysregulation, which has been mechanistically linked to the pathogenesis of major ophthalmic disorders. This review systematically elucidates the molecular architecture of mitophagy, focusing on its dual roles in disease progression and cytoprotection across glaucoma, age-related macular degeneration (AMD), and diabetic retinopathy (DR). By integrating mechanistic insights with therapeutic implications, we not only delineate conserved regulatory pathways (e.g., *PINK1* [PTEN-induced kinase 1]*/*Parkin*, BNIP3* [BCL2/adenovirus E1B 19 kDa interacting protein 3]*, FUNDC1* [FUN14 domain containing 1]) but also propose a roadmap for targeting mitophagic checkpoints through precision pharmacology and combinatorial regimens. Our synthesis underscores the urgency of translating mitophagy modulation into clinical strategies to address unmet needs in retinal degenerative diseases.

## 1. Introduction

Mitochondria are essential organelles central to cellular energy metabolism, regulating ATP synthesis, calcium homeostasis, redox balance, and apoptosis. When mitochondrial function is impaired, it can lead to severe cellular consequences, often culminating in cell death. In the retina, photoreceptor cells, retinal pigment epithelial (RPE) cells, and Müller glial cells are particularly rich in mitochondria, making them especially susceptible to mitochondrial dysfunction.^[1]^ Autophagy, a highly conserved cellular process in eukaryotes, plays a vital role in maintaining intracellular homeostasis by mediating the degradation of aggregated proteins and damaged organelles.^[2]^ Mitophagy is primarily regulated by the *PINK1/*Parkin signaling pathway, along with other key mechanisms involving factors such as *BNIP3* and *FUNDC1*.^[3-5]^ Emerging evidence underscores the critical role of mitophagy in the pathogenesis of major ocular disorders, including glaucoma, age-related macular degeneration (AMD), diabetic retinopathy (DR), and retinitis pigmentosa (RP). As retinal diseases continue to rise globally, understanding and targeting mitophagy could provide transformative therapeutic avenues. Investigating the molecular mechanisms underlying mitophagy and its involvement in these diseases is essential for understanding their pathological processes and laying the foundation for novel therapeutic approaches. This review provides a detailed summary of the core mechanisms of mitophagy, highlights its roles and recent research progress in various ocular diseases, and evaluates its potential as a therapeutic target. Collectively, these observations underscore the interplay between oxidative stress and mitophagy in retinal pathology. Under hyperglycemic conditions, excess reactive oxygen species (ROS) compromise mitochondrial integrity and trigger mitophagy-mediated antioxidant protection, which restores mitochondrial function and limits retinal injury (see Section 5.3 for disease-specific details).

## 2. Literature search and inclusion criteria

### 2.1. Systematic search strategy

To comprehensively identify relevant literature on mitophagy in ophthalmic pathologies, we conducted systematic searches in 3 major electronic databases: PubMed, Web of Science (Core Collection), and Embase (via OvidSP). The search was performed for articles published from January 1, 2000 to June 30, 2025. This broad timeframe (spanning over 25 years) was chosen to capture the foundational research on mitophagy mechanisms as well as the most recent advancements in its role in eye diseases, ensuring a comprehensive historical and contemporary perspective. While the core focus was on the latest findings, no specific start date cutoff was applied to ensure inclusion of seminal early works.

### 2.2. Search strings

The search strategy employed a combination of keywords and Medical Subject Headings (MeSH)/Emtree terms related to the core concepts: mitophagy AND ophthalmic diseases/ocular pathologies AND specific diseases (glaucoma, age-related macular degeneration (AMD), diabetic retinopathy (DR)). Boolean operators (AND, OR) were used to combine terms within and between concepts. The search strings were adapted to the specific syntax of each database. An example of the search strategy used in PubMed is provided below: `(“mitophagy”[MeSH Terms] OR “mitophagy”[Title/Abstract])` AND `(“eye diseases”[MeSH Terms] OR “ophthal”[Title/Abstract] OR “ocular”[Title/Abstract] OR “retina”[Title/Abstract] OR “glaucoma”[MeSH Terms] OR “glaucoma”[Title/Abstract] OR “macular degeneration”[MeSH Terms] OR “age-related macular degeneration”[Title/Abstract] OR “AMD”[Title/Abstract] OR “diabetic retinopathy”[MeSH Terms] OR “diabetic retinopathy”[Title/Abstract] OR “DR”[Title/Abstract])`.

Similar strategies were constructed for Web of Science and Embase, utilizing their respective thesauri and field tags (e.g., TS = [topic] in WoS). The complete search strategies for all databases are detailed in Supplementary Material S1, Supplemental Digital Content.

### 2.3. Inclusion criteria

Studies were included if they met the following criteria: Population/model: Studies investigating mitophagy in the context of glaucoma, AMD, DR, or related ocular tissues/cells (e.g., retinal ganglion cells [RGCs], retinal pigment epithelium [RPE], Müller cells, photoreceptors, corneal epithelium).

This included: In vitro studies using relevant human or animal cell lines (e.g., ARPE-19, RGC-5, Müller cell lines, corneal epithelial cell lines).

In vivo studies using established animal models of the diseases (e.g., rodent models: mice, rats; zebrafish models).

Ex vivo studies using human or animal ocular tissues (e.g., donor retinas, surgical specimens).

Clinical studies (observational or interventional) in human patients.

#### 2.3.1. Intervention/exposure

Studies examining the molecular mechanisms of mitophagy, its regulation, dysregulation, or modulation (genetic, pharmacological, or other means) in the specified ocular diseases or tissues.

#### 2.3.2. Outcome

Studies reporting on mitophagy activity (e.g., flux markers like LC3-II/I, p62/SQSTM1 degradation, mitophagosome counts, PINK1/Parkin activation, BNIP3/FUNDC1 expression), its functional consequences (e.g., cell survival/death, oxidative stress, inflammation, vascular changes, visual function), or its therapeutic targeting.

#### 2.3.3. Study design

Original research articles (basic science, translational, clinical), systematic reviews, and meta-analyses.

Comprehensive review articles were also included for background and contextual understanding, although primary research was prioritized for mechanistic and therapeutic synthesis.

### 2.4. Exclusion criteria

Studies were excluded based on:

Study type: Conference abstracts, editorials, letters (without original data), case reports (unless providing unique mechanistic insights), non-peer-reviewed publications (e.g., preprints not subsequently published in peer-reviewed journals).

Relevance: Studies not primarily focused on mitophagy in the specified ocular pathologies or relevant cell/tissue types. Studies solely on general autophagy without a specific focus on mitophagy were excluded unless they contained significant mitophagy-related data relevant to our scope.

Language: No language restrictions were applied during the initial search. Titles and abstracts of non-English articles were translated using automated tools (e.g., Google Translate) to assess relevance. If deemed relevant, the full text was translated for detailed evaluation.

**Figure 1.**
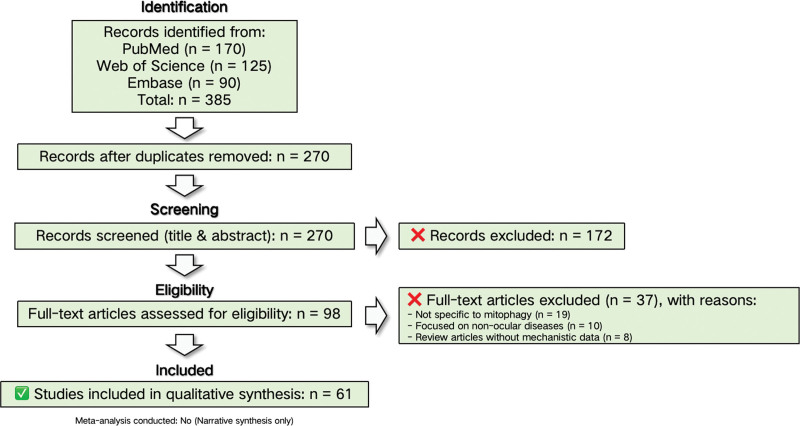
Flow diagram of study selection process. A total of 385 records were identified through systematic searches of PubMed (n = 170), Web of Science (n = 125), and Embase (n = 90). After removing 115 duplicate records, 270 unique titles and abstracts were screened, of which 172 were excluded for irrelevance. Ninety‑eight full‑text articles were assessed for eligibility; 37 were excluded (19 not specific to mitophagy, 10 focused on non‑ocular pathologies, and 8 lacked mechanistic data). Finally, 61 studies were included in the qualitative synthesis. No meta‑analysis was performed.

Screening and selection process: Search results from all databases were exported into a reference management software (e.g., Zotero), and duplicates were removed. Two independent reviewers (initials or roles, e.g., YH and JF) screened the titles and abstracts of all retrieved records against the inclusion/exclusion criteria. Full texts of potentially eligible articles. Full-text articles of potentially eligible studies were retrieved and assessed against the inclusion and exclusion criteria. The selection process is detailed in the flow diagram (Fig. 1). Furthermore, a quality assessment of the included studies using ROB and GRADE tools is summarized in Table S2, Supplemental Digital Content. This study is a literature-based review and did not involve any human participants or animals. Therefore, ethical approval and informed consent were not applicable.

## 3. Fundamental physiological characteristics of mitochondria and mitophagy

Mitochondria are essential and evolutionarily conserved organelles in eukaryotic cells, characterized by a double-membrane structure comprising an outer membrane, an inner membrane, and an intermembrane space, with the matrix enclosed by the inner membrane.^[6,7]^ Commonly known as the “powerhouse of the cell,”^[8]^ mitochondria generate adenosine triphosphate (ATP) by utilizing oxygen and glucose, supplying vital energy for cellular processes and regulating key aspects of energy metabolism. The integrity of mitochondrial function is critical for maintaining cellular energy production and metabolic efficiency.^[9]^ However, with aging and the onset of diseases, mitochondrial ATP production gradually declines.^[10]^ In addition to energy production, mitochondria regulate calcium homeostasis, control ATP synthesis, and modulate ROS production through dynamic fission and fusion processes. These functions are tightly regulated to support intermediary metabolism and programmed cell death, thereby maintaining cellular homeostasis.^[11,12]^ Mitochondrial dysfunction often impairs metabolic flexibility, contributing to the onset and progression of various diseases.^[12]^ Beyond their role in cellular energy metabolism, mitochondria also contribute to overall metabolic homeostasis through intercellular interactions. For example, some cells can meet their metabolic needs by acquiring mitochondria from donor cells.^[13]^ Moreover, donor cells help maintain mitochondrial quality control by releasing damaged mitochondria via extracellular vesicles (EVs). This process allows brown adipocytes and cardiomyocytes to transfer dysfunctional mitochondria to tissue-resident macrophages for degradation, thereby ensuring mitochondrial health.^[14]^

Mitophagy, a selective autophagic process, is primarily responsible for identifying and eliminating damaged or dysfunctional mitochondria, preserving mitochondrial network quality, and maintaining cellular homeostasis. The mechanism of mitophagy involves several key steps and molecular regulations, with the *PINK1/*Parkin pathway being one of the most crucial.^[15]^ Under normal conditions, *PINK1* is transported to the mitochondria, where it is degraded in the inner membrane. However, when mitochondrial membrane potential is reduced or mitochondria are damaged, *PINK1* stabilizes on the depolarized mitochondrial outer membrane (OMM), initiating a phosphorylation cascade that recruits and activates Parkin E3 ubiquitin ligase activity.^[16]^ Activated Parkin, as an E3 ubiquitin ligase, ubiquitinates various mitochondrial membrane proteins, which are then recognized by autophagy receptors such as p62/Sequestosome,^[17]^ NBR1,^[18]^ nuclear dot protein 52,^[19]^ OPTN (optineurin),^[20]^ and TAX1BP1 (T-cell lymphoma invasion and metastasis 1-binding protein 1).^[21]^ These receptors facilitate the conjugation of damaged mitochondria with autophagosomes, which are then delivered to lysosomes for degradation. This process ensures the health and functional integrity of the mitochondrial network, maintaining the balance of mitochondrial quality and quantity within the cell and supporting essential physiological functions, including cellular energy metabolism. For example, in the retina, mature photoreceptor cells, which have high metabolic demands, depend on mitophagy to remove damaged mitochondria rapidly and sustain normal function.^[22,23]^ In pathological conditions, such as diabetic retinopathy (DR), a hyperglycemic environment induces oxidative stress in Müller cells, leading to the accumulation of misfolded or unfolded proteins, which activate autophagic mechanisms. However, this process also results in lysosome-mediated autophagic dysfunction, contributing to increased cell apoptosis and excessive secretion of vascular endothelial growth factor (VEGF).^[24,25]^

Mitophagy is vital for maintaining mitochondrial quality and cellular energy metabolism, and its dysfunction is closely associated with a variety of diseases. A comprehensive investigation into the regulatory mechanisms of mitophagy, particularly its involvement in ocular diseases, will provide a theoretical foundation and practical insights for developing novel therapeutic strategies.

## 4. Autophagy and signaling pathways in mitophagy

**Figure 2.**
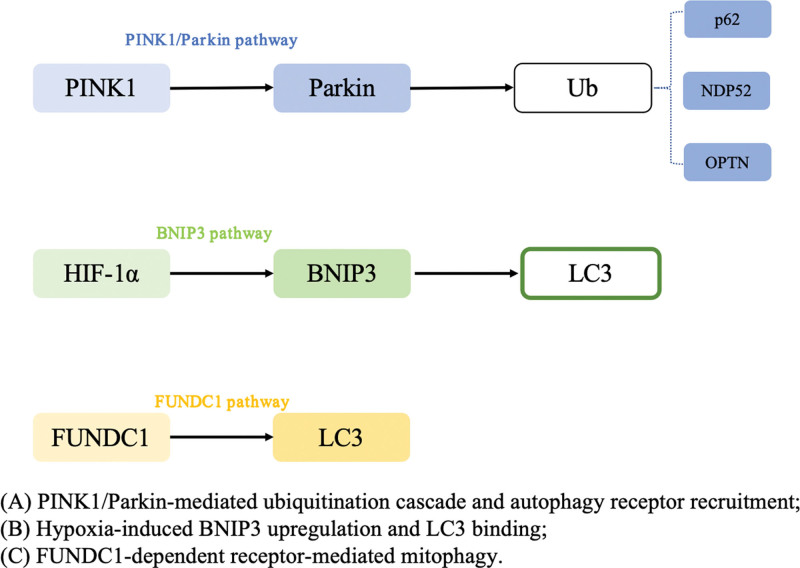
Schematic representation of mitophagy signaling pathways in ocular tissues. (A) PINK1/Parkin-mediated cascade: Mitochondrial depolarization stabilizes PINK1 on the outer mitochondrial membrane (OMM), recruiting and activating the E3 ligase Parkin. Parkin ubiquitinates OMM substrates, which are recognized by autophagy receptors (p62, NDP52, OPTN; TBK1‑phosphorylated) that bind LC3 via their LC3‑interacting regions (LIRs) to initiate mitophagosome formation. (B) Hypoxia‑induced BNIP3 pathway: HIF‑1α upregulates BNIP3 under stress, and BNIP3 directly engages LC3 through its LIR motif to target damaged mitochondria for autophagic clearance. (C) FUNDC1‑dependent receptor pathway: FUNDC1 on the OMM acts as a mitophagy receptor, binding LC3 via its LIR to promote selective removal of dysfunctional mitochondria. Orange arrows denote cross‑talk between pathways (e.g., Parkin‑mediated modulation of HIF‑1α/BNIP3 expression and BNIP3 influence on FUNDC1 activity). *BNIP3* = BCL2/adenovirus E1B 19 kDa interacting protein 3, *FUNDC1* = FUN14 domain containing 1, HIF-1α = hypoxia-inducible factor 1-alpha, LC3 = microtubule-associated protein 1A/1B-light chain 3, LIR = LC3-interacting regions, NDP52 = nuclear dot protein 52, OMM = outer mitochondrial membrane, OPTN = optineurin, Parkin = E3 ubiquitin ligase Parkin, *PINK1* = PTEN-induced kinase 1.

The PINK1/Parkin-mediated cascade and receptor‑mediated pathways are summarized in the schematic diagram below (Fig. 2).

### 4.1. PINK1/Parkin pathway

Mitophagy is a crucial process for cellular mitochondrial quality control, primarily regulated by the *PINK1/*Parkin pathway. This pathway facilitates autophagosome formation to eliminate damaged mitochondria by degrading *PINK1* in healthy mitochondria and stabilizing it at the outer membrane of damaged mitochondria through Parkin activation. The process involves various protein modifications and collaboration with other mitochondrial quality control mechanisms.

Han Zhao et al demonstrated that the expression of *PINK1* and Parkin was elevated in the lacrimal gland of senescent mice following intraperitoneal injection of rapamycin or Mdivi-1 (mitochondrial division inhibitor 1) in 6-week-old and 12-month-old male C57BL/6J mice. Moreover, rapamycin treatment further upregulated their expression. Similarly, D’Amico et al^[26]^ found that in high glucose (HG)-treated Müller cells, the LC3 (microtubule-associated protein 1A/1B-light chain 3)II/LC3I ratio was increased while the expression of p62 was decreased, indicating enhanced mitophagy. When autophagic flow was blocked, p62 accumulated, suggesting the regulation of mitophagy by the *PINK1/*Parkin pathway. Additionally, a study by Zhang et al^[27]^ revealed that the deletion of PCSK9 promotes *PINK1*-Parkin pathway-mediated mitophagy, reduces mtDNA release, and mitigates injury by inhibiting the cGAS-STING pathway and NLRP3 inflammasome activation, which are otherwise induced by the inhibition of *PINK1*-Parkin-mediated mitophagy. Zhang et al^[28]^ showed that Sirt3 influences the *PINK1/*Parkin pathway by regulating FOXO3a (Forkhead box O3). Under high glucose conditions, reduced Sirt3 expression in retinal pigment epithelium (RPE) cells weakens the FOXO3a/*PINK1*-Parkin-mediated mitophagy pathway, resulting in an increase in intracellular ROS levels. In contrast, Sirt3 overexpression activates this pathway and inhibits apoptosis. Finally, Li et al^[29]^ demonstrated that TBK1 (TANK-binding kinase 1) plays a key regulatory role in *PINK1/*Parkin-mediated mitophagy by phosphorylating autophagy receptors such as OPTN and nuclear dot protein 52, thereby promoting the binding of these receptors to mitochondria and enhancing mitophagy.

### 4.2. BNIP3-mediated pathway

Factors like *BNIP3* and *FUNDC1* play crucial roles in hypoxic conditions, metabolic reprogramming, cellular injury, and inflammatory responses by regulating mitochondrial function and maintaining mitochondrial dynamics, thereby influencing cell fate and pathophysiological processes.

In the *BNIP3*-mediated pathway, Sun et al^[30]^ found that *BNIP3* knockdown decreased the expression of the autophagy marker protein LC3B-II, increased the expression of associated proteins such as SQSTM1, and reduced the number of mitochondrial autophagosomes. Conversely, *BNIP3* overexpression produced the opposite effect. Sun et al^[4]^ demonstrated that in mice, Pptc7 knockdown led to elevated levels of *BNIP3* and *NIX*, causing hyperactivation of mitophagy, mitochondrial loss, and perinatal death; however, Nix knockdown rescued this phenotype. Zhang et al^[31]^ found that *JMJD5* overexpression inhibited apoptosis by upregulating hypoxia-inducible factor 1-alpha, promoting *BNIP3* expression, and enhancing the expression of autophagy-related proteins, such as mitochondrial LC3-I. In contrast, knocking down *BNIP3* aggravated apoptosis, inhibited mitophagy, and diminished the protective effects of *JMJD5*. In the *FUNDC1*-mediated pathway, Bai et al^[5]^ showed that upregulation of *FUNDC1* expression significantly impacted mitochondrial function by regulating the formation of MAMs, maintaining mitochondrial calcium homeostasis, and promoting mitochondrial fragmentation. In contrast, downregulation or deletion of *FUNDC1* disrupted MAM formation, reduced mitochondrial calcium levels, and caused mitochondrial morphological abnormalities. Dong et al^[32]^ found that in the human lens epithelial cell line SRA01/04, *FUNDC1* promoted apoptosis by increasing the expression of Bax and cleaved Caspase-3, and enhanced autophagy by increasing the LC3-II/LC3-I ratio.

**Figure 3.**
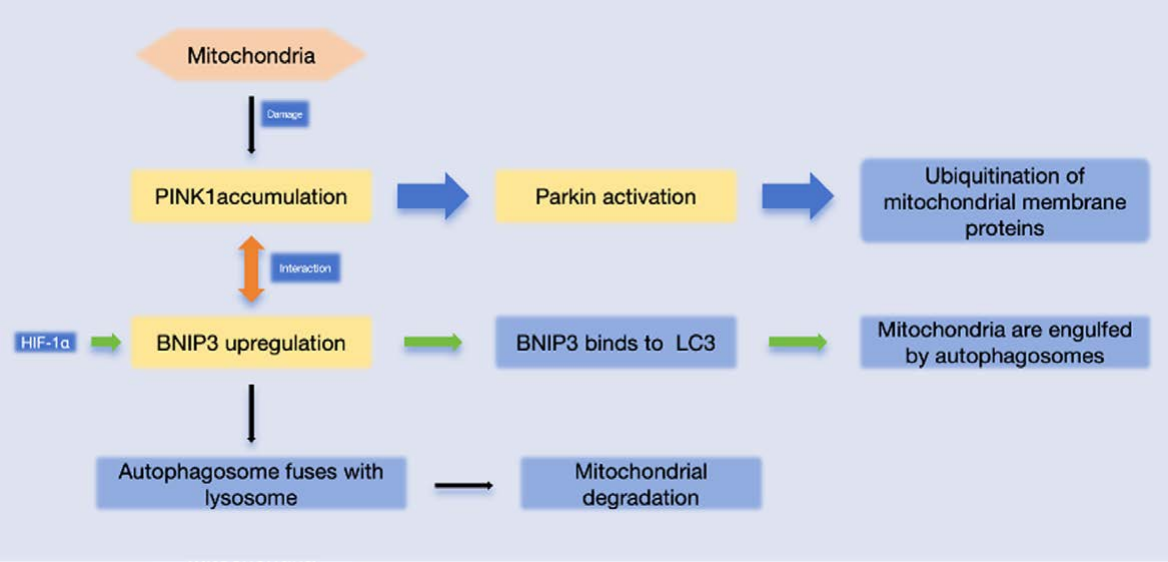
Mitophagic signaling network in ocular tissues. Blue: Used for the *PINK1/*Parkin pathway, indicating the dominant mitochondrial autophagy pathway. Green: Used for the *BNIP3* pathway, representing the regulation of mitochondrial autophagy under hypoxic or stress conditions. Orange: Indicate the interaction between the *PINK1/*Parkin and *BNIP3* pathways. Yellow highlights key steps, such as the initiation steps of the *PINK1/*Parkin pathway or the upregulation of *BNIP3*. Upon mitochondrial damage, *PINK1* (PTEN-induced kinase 1) accumulates on the outer mitochondrial membrane. This accumulation leads to the activation of Parkin, which ubiquitinates mitochondrial membrane proteins. *BNIP3* is upregulated via interaction with HIF-1α, and *BNIP3* then binds to LC3, facilitating the engulfment of damaged mitochondria by autophagosomes. The autophagosomes subsequently fuse with lysosomes, leading to the degradation of the damaged mitochondria. This process ensures mitochondrial quality control and cellular homeostasis. *BNIP3* = BCL2/adenovirus E1B 19 kDa interacting protein 3, *FUNDC1* = FUN14 domain containing 1, HIF-1α = hypoxia-inducible factor 1-alpha, LC3 = microtubule-associated protein 1A/1B-light chain 3, Parkin = E3 ubiquitin ligase Parkin*, PINK1* = PTEN-induced kinase 1.

In summary, current studies have highlighted the crucial roles of key molecules such as *PINK1/*Parkin*, BNIP3*, and FUNDC1, as well as their involvement in mitophagy across various pathophysiological conditions. These findings offer new insights into the regulation of cellular metabolism, apoptosis, and inflammatory responses. Future research should focus on the following areas: first, elucidating the specific regulatory mechanisms of mitophagy in different cell types using multi-omics approaches; second, investigating the interaction between mitophagy and other cellular signaling pathways in greater detail to establish a theoretical foundation for integrated therapeutic strategies; and finally, closely linking basic research with clinical practice to develop precise regulatory tools for key molecules such as *PINK1*, *BNIP3*, and *FUNDC1*, with the aim of achieving personalized and targeted treatments for diseases (to provide a more integrated understanding, the mitophagic signaling network in ocular tissues is summarized in Fig. 3).

## 5. The role of mitophagy in major ophthalmic diseases

### 5.1. Mitophagy and glaucoma

Mitophagy dysfunction plays a critical role in the pathogenesis of glaucoma, contributing to several pathological processes, including oxidative stress, metabolic dysregulation, and ferroptosis, as well as the disruption of key signaling pathways, such as the *PINK1*-Parkin and TBK1-OPTN axes.

In a mouse model, Panda et al^[33]^ demonstrated that the dysfunction of the mitochondrial electron transport chain in retinal ganglion cells (RGCs) induces oxidative stress, modulated via the 14-3-3 proteins and its regulatory impact on TAK-1, PPM1B, and NF-κB (Nuclear factor kappa-light-chain-enhancer of activated B cells). Liang et al^[34]^ further observed that key mitophagy receptors such as *BNIP3*, *NIX*, and *FUNDC1* show altered expression patterns in glaucomatous progression, affecting LC3 interactions and autophagic efficiency. Notably, Dai et al^[35]^ demonstrated that in chronic hypertensive glaucoma rats, overexpression of Parkin by AAV2 vectors significantly protected RGCs, restored mitophagic flux (measured by LC3‑II/I ratio and mitophagosome count), and improved mitochondrial health under elevated IOP.

In high-glucose-treated human Müller cells, Zhang et al^[36]^ found that promoting *PINK1/*Parkin-mediated mitophagy significantly protected RGCs. PARKIN overexpression mitigated RGC loss. Of note, OPTN mutations, which impair its function as a mitophagy receptor, have been directly associated with primary open-angle glaucoma (POAG) in human studies.^[37]^

Chen et al^[38]^ revealed that NCOA4 (nuclear receptor coactivator 4)-mediated ferritin autophagy alters intracellular Fe2+ levels, indirectly modulating mitochondrial status and mitophagy. D’Urso et al^[39]^ confirmed that mutations in the OPTN (e.g., E50K, M98K, H486R) impair mitophagic flux and disrupt NF-κB signaling, leading to axonal transport deficits and enhanced ROS accumulation.

Collectively, both preclinical and clinical findings converge on mitophagy dysfunction as a key pathological mechanism in glaucoma. While animal models have elucidated upstream molecular pathways, further validation in patient-derived cells and clinical contexts is essential.

### 5.2. Mitophagy and age-related macular degeneration (AMD)

Recent studies have highlighted the crucial role of mitophagy in age-related macular degeneration (AMD). Several investigations across murine models and human RPE cells have demonstrated that phytochemicals, specific light wavelengths, and modulation of mitophagy-related signaling pathways can enhance RPE cell function and mitigate AMD progression.

In a human RPE degeneration model, Pinelli et al^[40]^ demonstrated that overexpression of NLRX1 (NOD-like receptor X1) reversed autophagy suppression and oxidative damage. Fisher et al^[41]^ found that FCCP stimulation activated mitochondrial fission proteins and mitophagy markers, though the response was blunted in AMD patient-derived RPE cells. In contrast, CoCl_2_ treatment mildly enhanced mitophagy under experimental AMD conditions. In a zebrafish RPE model, Zheng et al^[42]^ showed that exposure to quantum‑dot–induced oxidative stress triggered ferroptosis and mitophagy in RPE cells, illustrating conserved mitophagic mechanisms in non‑mammalian vertebrates.

Lewis Luján et al^[43]^ activated Sirt1 (Sirtuin 1),^[44]^ AMPK (adenosine monophosphate-activated protein kinase),^[45]^ Nrf2 (nuclear factor erythroid 2-related factor 2),^[46]^ and PPARα (peroxisome proliferator-activated receptor alpha)^[47]^ pathways in AMD animal models and observed enhanced mitophagy and reduced ROS. These findings, supported by patient and animal studies, point to dysregulated mitophagy as a pathogenic feature in AMD.

In clinical samples, PGC-1α (peroxisome proliferator-activated receptor gamma coactivator 1-alpha) expression was notably diminished, correlating with impaired mitochondrial biogenesis and antioxidant defense.^[48]^ Moreover, elevated LCN2 levels inhibited autophagic flux in AMD-affected RPE cells.^[38]^ Together, these observations underscore the translational relevance of mitophagy modulation in AMD therapy.

In summary, current studies suggest that the regulation of mitophagy offers novel insights into potential treatments for age-related macular degeneration (AMD). Future research should further explore the possibility of improving RPE cell function by enhancing the autophagic pathway and antioxidant defense mechanisms, particularly in the context of clinical translation. This approach could lead to more effective therapeutic strategies for AMD patients. Additionally, future studies should not only focus on developing new drugs and nutritional interventions but also aim to refine the precise regulation of mitochondrial function restoration and autophagy pathways, thereby opening up new possibilities for the early diagnosis and personalized treatment of AMD.

### 5.3. Mitophagy and diabetic retinopathy (DR)

Mitophagy is critically implicated in diabetic retinopathy. Under hyperglycemic conditions, mitochondrial damage accumulates due to ROS generation and impaired autophagy. As introduced earlier, mitophagy-mediated antioxidant protection plays a key role in reestablishing mitochondrial homeostasis.

Chen et al^[38]^ demonstrated that in high-glucose environments, abnormal *PINK1* activation or Parkin suppression impairs mitophagic clearance. In a diabetic mouse model, Zhang et al^[28]^ showed that Sirt3 activated the *PINK1*-Parkin axis and protected against retinal oxidative damage. Human RPE cell data confirmed the relevance of this mechanism in vitro.

Devi et al^[49]^ highlighted that the TXNIP (Thioredoxin-interacting protein) inhibits mitophagy through the mitochondria-lysosome axis, promoting cellular apoptosis. However, TXNIP knockdown restored mitophagic flux. Similarly, overexpression of Sirt3 in high-glucose RPE cells

Activated the FOXO3a/PINK1-Parkin axis and prevented cell death.^[50]^ In cultured human RPE cells, trehalose pre‑treatment upregulated transcription factor EB‑mediated autophagy and protected against hydroquinone‑induced oxidative damage – an effect dependent on autophagic flux, supporting its potential to preserve mitochondrial health in a diabetic milieu.^[51]^

In parallel, Notoginsenoside R1 (NGR1) promoted mitophagy and reduced intracellular ROS in both cellular and animal models.^[52]^

While these findings highlight the therapeutic potential of modulating mitophagy in DR, most data remain preclinical. Clinical confirmation of these pathways in patient tissue or trials is still pending.

### 5.4. Mitophagy and other ocular diseases

In the context of other ophthalmic pathologies, Intartaglia et al^[53]^ investigated the role of mitophagy in retinal pigment epithelium (RPE) homeostasis. Their findings demonstrate that mitophagy serves as a critical quality control mechanism for mitochondrial integrity in RPE cells. Under physiological conditions, this process is tightly regulated through the AMPK-mTOR signaling axis, where AMPK modulates mTORC1 activity in response to metabolic or oxidative stress conditions, thereby maintaining mitochondrial fidelity. During mitochondrial dysfunction, the *PINK1*-Parkin pathway orchestrates selective autophagic clearance by ubiquitinating damaged organelles and targeting impaired mitochondria for lysosomal degradation. Ayilam Ramachandran et al^[54]^ reviewed that in corneal epithelial cells subjected to hyperosmotic stress (a dry-eye model), mitophagy activation was critical for reducing inflammatory cytokine release and epithelial apoptosis, underscoring mitophagy’s protective role across corneal pathologies.

**Figure 4.**
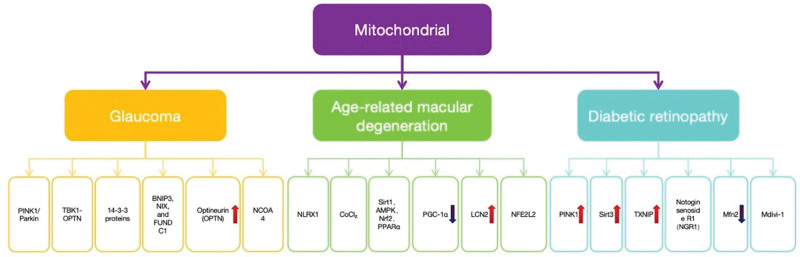
The diagram shows the involvement of mitochondrial autophagy in 3 major eye diseases: glaucoma, age-related macular degeneration (AMD), and diabetic retinopathy. The pathways and molecules involved in mitochondrial regulation for each disease are depicted as follows: Glaucoma: Key molecules involved: *PINK1/*Parkin, TBK1-OPTN, 14-3-3 proteins, *BNIP3*, *NIX*, and *FUNDC1*, as well as OPTN and NCOA4 (nuclear receptor coactivator 4). AMD: Key molecules and signaling pathways include NLRX1 (NOD-like receptor X1), CoCl_2_, Sirt1 (Sirtuin 1), AMPK (adenosine monophosphate-activated protein kinase), Nrf2 (nuclear factor erythroid 2-related factor 2), PPARα (peroxisome proliferator-activated receptor alpha), PGC-1α (peroxisome proliferator-activated receptor gamma coactivator 1-alpha), LCN2 (lipocalin 2), and NFEL2L2 (nuclear factor, erythroid 2 like 2). Diabetic retinopathy: Important molecules include PINK1, Sirt3, TXNIP (thioredoxin-interacting protein), NGR1 (notoginsenoside R1), and Mfn2 (mitofusin 2), along with the autophagy modulator Mdivi-1 (mitochondrial division inhibitor 1). Arrows indicate the regulation or involvement of specific proteins and signaling pathways in mitochondrial autophagy and disease progression. AMPK = adenosine monophosphate-activated protein kinase, *BNIP3* = BCL2/adenovirus E1B 19 kDa interacting protein 3, CoCl₂ = cobalt chloride, *FUNDC1* = FUN14 domain containing 1, LCN2 = lipocalin 2, Mdivi-1 = mitochondrial division inhibitor 1, Mfn2 = mitofusin 2, NCOA4 = nuclear receptor coactivator 4, NFE2L2 = nuclear factor, erythroid 2 like 2, NGR1 = notoginsenoside R1, NLRX1 = NOD-like receptor X1, Nrf2 = nuclear factor erythroid 2-related factor 2, OPTN = optineurin, Parkin = E3 ubiquitin ligase Parkin, *PINK1* = PTEN-induced kinase 1, PGC-1α = peroxisome proliferator-activated receptor gamma coactivator 1-alpha, PPARα = peroxisome proliferator-activated receptor alpha, Sirt1 = Sirtuin 1, TBK1 = TANK-binding kinase 1, TXNIP = thioredoxin-interacting protein.

Ayilam Ramachandran et al^[54]^ demonstrated that hyperosmotic stress and inflammatory mediators activate mitophagy in corneal epithelial cells during dry eye disease pathogenesis. This compensatory mechanism attenuates inflammatory cascades and preserves cellular equilibrium through selective degradation of dysfunctional mitochondria. Pharmacological modulation studies identified specific mitophagy inducers (LYN-1604, trehalose, melatonin) that significantly ameliorate clinical manifestations of xerophthalmia, whereas autophagy suppression exacerbates pathological manifestations. Furthermore, in Fuchs endothelial corneal dystrophy, researchers observed concomitant upregulation of mitophagic activity with downregulation of mitochondrial fusion protein MFN2 and fragmentation of mitochondrial networks in corneal endothelial cells, suggesting mitophagy’s dual regulatory role in disease pathogenesis. These findings collectively establish mitophagy as a central regulatory mechanism across corneal pathologies (as illustrated in Fig. 4, distinct mitophagy-related signaling mechanisms are implicated in the pathogenesis of glaucoma, AMD, and diabetic retinopathy).

## 6. Synthesis and future perspectives

Recent studies on mitophagy in ophthalmic pathologies have elucidated its pivotal regulatory mechanisms in glaucoma, age-related macular degeneration (AMD), and diabetic retinopathy (DR), providing critical insights for therapeutic target identification. As the cornerstone of mitochondrial quality surveillance, mitophagic flux is coordinated through evolutionarily conserved mechanisms (e.g., PINK1/Parkin pathway, receptor-mediated clearance, and lipid signaling), and plays a fundamental role in metabolic adaptation and cellular protection. Integrating mitophagy with disease-specific pathogenic pathways offers a promising strategy to restore mitochondrial homeostasis.

### 6.1. Translational landscape: opportunities and challenges

Recent advances have demonstrated the translational potential of targeting mitophagy in ocular diseases. A randomized controlled trial showed that high-dose nicotinamide (vitamin B_3_) significantly improved retinal ganglion cell function and visual field stability in glaucoma patients by enhancing mitochondrial quality control.^[55]^ Preclinical work in murine models has further validated these findings: nicotinamide riboside (NR), a nicotinamide adenine dinucleotide precursor, preserved photoreceptor structure and function by activating PINK1/Parkin-mediated mitophagy.^[56]^ Similarly, melatonin and its synthetic analogs have been reported to reduce oxidative damage, modulate autophagy, and lower intraocular pressure in glaucoma and AMD animal models.^[57]^ These studies underscore the feasibility of mitophagy modulation as a therapeutic strategy (Table 1 summarizes the leading mitophagy‑targeted therapeutic candidates, their molecular targets, and development status).

**Table 1:** Therapeutic candidates targeting mitophagy in ophthalmic diseases, with their mechanisms and current development status.

Candidate	Targeted mitophagy pathway	Model or trial status	References
Nicotinamide (vitamin B_3_)	PINK1/Parkin activation	Phase II clinical trial in glaucoma patients	Hui et al^[55]^
Nicotinamide riboside	NAD^+^ precursor → PINK1/Parkin	Preclinical (mouse retinal degeneration)	Zhang et al^[56]^
Melatonin analogs	Autophagy/mitophagy modulation	Preclinical (glaucoma and AMD models)	Alkozi et al^[57]^
Notoginsenoside R1	PINK1-dependent mitophagy	Preclinical diabetic retinopathy (cells and mice)	Zhou et al^[52]^
Trehalose	TFEB-mediated autophagy	In vitro human RPE study	Abokyi et al^[51]^
LYN-1604	OPTN/BNIP3-dependent mitophagy	Preclinical dry-eye model	Ramachandran et al^[54^^]^

AMD = age-related macular degeneration, *BNIP3* = BCL2/adenovirus E1B 19 kDa interacting protein 3, NAD = nicotinamide adenine dinucleotide, OPTN = optineurin, Parkin = E3 ubiquitin ligase Parkin, *PINK1* = PTEN-induced kinase 1, TFEB = transcription factor EB.

### 6.2. Limitations

Clinical translation of mitophagy‑targeted treatments for posterior‑segment ocular disorders still encounters several major roadblocks. First, the blood–retinal barrier remains a formidable obstacle for most small‑molecule drugs, effectively demanding next‑generation delivery platforms. Next is the biomarker dilemma: reliable, noninvasive methods to assess mitophagic activity in living patients are lacking, making it difficult to determine whether a given therapy is engaging its intended target. Moreover, the bulk of preclinical evidence has been derived from rodent models – and rodent mitochondrial behavior does not always mirror that of humans – so promising laboratory findings can lose potency in clinical settings. Lastly, conditions such as AMD and DR exhibit significant inter‑patient biological variability, underscoring the importance of patient stratification in future trials. Ultimately, overcoming these challenges will depend on additional early‑phase human studies and comprehensive tissue analyses to validate the feasibility of mitophagy modulation in patients.

### 6.3. Future directions

Future directions should prioritize the development of ocular-specific delivery systems, quantitative mitophagy imaging techniques, and standardized preclinical models that recapitulate human retinal disease phenotypes. In addition, integrating mitophagy modulators with existing standard-of-care therapies may offer additive or synergistic benefits. Ultimately, multicenter collaborative studies and early-phase clinical trials will be critical to move mitophagy-targeted treatments from bench to bedside.

Supplemental digital content “Table S1” is available for this article.

## Author contributions

**Conceptualization:** Hongsheng Bi, Yibo Han.

**Investigation:** Xiaoqi Gong, Guodong Tang, Yixue Yin, Jing Li, Yuxi Liu, Jun Zhang.

**Supervision:** Hongsheng Bi, Jike Song.

**Writing – original draft:** Yibo Han.

**Writing – review & editing:** Jiaojiao Feng, Jike Song, Hongsheng Bi.






